# Identification and characterization of a novel antifungal compound tubeimoside I targeting cell wall

**DOI:** 10.1128/spectrum.04047-23

**Published:** 2024-04-23

**Authors:** Qiannan Liu, Zhiqi Zhong, Shunxin Zheng, Yunzhuo Chu, Norihiro Sakamoto, Takayoshi Kuno, Yue Fang

**Affiliations:** 1 Department of Microbial and Biochemical Pharmacy, School of Pharmacy, China Medical University, Shenyang, Liaoning Province, China; 2 Department of Laboratory Medicine, The First Affiliated Hospital of China Medical University, Shenyang, Liaoning Province, China; 3 Division of Food and Drug Evaluation Science, Kobe University Graduate School of Medicine, Kobe, Japan; Agroscope, Nyon, Switzerland

**Keywords:** tubeimoside I (TBMS1), antifungal activity, cell wall, *Schizosaccharomyces pombe*

## Abstract

Due to fungal diseases that threaten immunocompromised patients, along with the limited availability of antifungal agents, there is an urgent need for new antifungal compounds to treat fungal infections. Here, we aimed to identify potential antifungal drugs from natural products using the fission yeast *Schizosaccharomyces pombe* as a model organism since it shares many features with some pathogenic fungi. Here, we identified tubeimoside I (TBMS1), an extract from Chinese herbal medicine, that showed strong antifungal activity against *S. pombe*. To gain insight into the underlying mechanism, we performed transcriptomics analyses of *S. pombe* cells exposed to TBMS1. A significant proportion of the differential expressed genes were involved in cell wall organization or biogenesis. Additionally, TBMS1 treatment of *S. pombe* cells resulted in pleiotropic phenotypes, including increased sensitivity to β-glucanase, enhanced calcineurin activity, translocation of GFP-Prz1 to the nucleus, as well as enhanced dephosphorylation of Prz1, suggesting that TBMS1 disrupted cell wall integrity of *S. pombe* cells. Notably, calcofluor staining showed that abnormal deposits of cell wall materials were observed in the septum and cell wall of the TBMS1-treated cells, which were further corroborated by electron microscopy analysis. We also found that oxidative stress might be involved in the antifungal action of TBMS1. Moreover, we confirmed the antifungal activities of TBMS1 against several clinical isolates of pathogenic fungi. Collectively, our findings suggest that TBMS1, a novel antifungal compound, exerts its antifungal activity by targeting cell walls, which may pave the way for the development of a new class of antifungals.

**IMPORTANCE:**

Fungal infections pose a serious threat to public health and have become an emerging crisis worldwide. The development of new antifungal agents is urgently needed. Here, we identified compound tubeimoside I (TBMS1) for the first time showing strong antifungal activity, and explored the underlying mechanisms of its antifungal action by using the model yeast *Schizosaccharomyces pombe*. Notably, we presented multiple evidence that TBMS1 exerts its antifungal activity through targeting fungal cell walls. Moreover, we verified the antifungal activities of TBMS1 against several pathogenic fungi. Our work indicated that TBMS1 may serve as a novel antifungal candidate, which provides an important foundation for designing and developing new cell wall-targeting agents for combating life-threatening fungal infections.

## INTRODUCTION

High morbidity and mortality caused by invasive fungal infections have become one of the serious public health issues worldwide over the last few decades, especially for immunocompromised patients such as AIDS, leukemia, transplant recipients, as well as super-aged people (1–3). The majority of human fungal infections are caused by species of the *Candida genus*, including *Candida albicans*, while invasive fungal infections caused by non-*albicans Candida* species and other less-common emerging species are increasing in prevalence due to the cooccurrence of lethal fungal infections with coronavirus disease 2019 (COVID-19), highlighting the continued threat fungal pathogens pose to human health (4–6). Due to the limited availability of antifungal agents for combating an increasing number of fungal infections, along with the emergence of fungal resistance against the commonly used antifungals, there is an urgent need for the development of novel antifungals to treat fungal infectious disease (7).

We have been screening the potential antifungal candidates and studying the molecular mechanisms underlying the actions of their antifungal activities by using the fission yeast *Schizosaccharomyces pombe* as a model organism, since it shares many features with pathogenic fungi and is amenable to genetic analysis. *S. pombe* is also an excellent organism for the identification of the molecular targets of various drugs since the major signaling pathways and processes involved in the cellular responses to cytotoxic agents are conserved between yeast and mammalian cells (8). Previously, we explored the underlying mechanism of the breast cancer therapeutic tamoxifen as an antifungal drug and identified the molecular target of its action by using *S. pombe* (9), since tamoxifen possesses antifungal properties as a good example of repurposed compounds developing for other therapeutic means (10).

Nowadays, natural products and their semisynthetic derivatives have been recognized as valuable sources for the discovery of novel bioactive compounds, and therapies developed from natural products have emerged since they present a series of advantages, such as lower costs and adverse reactions, easier access, as well as less negative impact on individual health (5, 11). In this study, we aimed to identify potential antifungal drugs from natural products by using *S. pombe*. Several interesting compounds with strong activities against *S. pombe* were identified, and here we focused on the compound tubeimoside I (TBMS1), which showed the highest antifungal activity against *S. pombe*.

TBMS1 is a natural compound extracted from the Chinese medicinal herb *Bolbostemma paniculatum* (Maxim) Franquet (Cucurbitaceae), which is traditionally used for the treatment of snake venoms and inflammation (12). Recent studies demonstrated that TBMS1 exerted potential anticancer effects in a variety of human cancers including lung cancer, ovarian cancer, colon cancer, liver cancer, gastric cancer, and so on (12–16). However, no studies have evaluated the ability of TBMS1 to inhibit the cell growth of fungi. Here, we identified TBMS1 as a novel antifungal candidate and characterized the underlying mechanisms, involving antifungal activity of TBMS1 in the model yeast to better understand modes of action. Moreover, we verified the antifungal activities of TBMS1 against several clinical isolates of pathogenic fungus from hospitalized patients with fungal infections. This study described the first demonstration of a conserved antifungal mechanism of TBMS1 not only in the non-pathogenic yeast *S. pombe* but also in the pathogenic fungi, indicating that TBMS1 may serve as a novel antifungal candidate to combat fungal infections.

## MATERIALS AND METHODS

### Strains and media

The *S. pombe* wild-type strain (*h^−^ leu1-32*), ∆*pmk1* deletion strain (*h^−^ leu1-32 ura4-D18 pmk1::ura4^+^*), and ∆*pek1*∆*mkh1* double deletion stain (*h^−^ leu1-32 ura4-D18 pek1::ura4^+^ mkh1::ura4^+^*) were used in this study. The reference strain of *C. albicans* (CPCC400616) was obtained from the China Pharmaceutical Culture Collection. The pathogenic fungi strains isolated from hospitalized patients with fungal infections in the First Affiliated Hospital of China Medical University are listed in Table 1. The complete medium yeast extract-peptone-dextrose (YPD), the minimal medium Edinburgh minimal medium (EMM), and the rich yeast extract with supplements were used as previously described (17, 18). The potato dextrose agar (PDA) medium was used to incubate the pathogenic fungi (19, 20). All chemicals and reagents were from commercial sources.

**TABLE 1:** *In vitro* antifungal activity of TBMS1^*a*^

Strains (number of strains)	MIC_50_ (μg/mL)		MIC_80_ (μg/mL)	
	FLZ	TBMS1	FLZ	TBMS1
*S.pombe*	32	4	128	8
*C. albicans* CPCC400616	0.5	32	1	64
*C. albicans* (3)	0.5–2	32–64	1–4	32–64
*C. krusei* (1)	32	16	32	32
*C. inconspicua* (1)	2	16	2	32
*C. tropicalis* (5)	4–128	32–64	32–256	64
*Cryptococcus* (1)	0.5	64	1	64
*Penicillium* (2)	16–64	32–64	≥256	32–64
*Trichoderma* (1)	0.5	32	32	32

^*a*^ FLZ, fluconazole; MIC, minimum inhibitory concentration.

### Determination of minimum inhibitory concentrations

To determine the minimum inhibitory concentrations (MICs) of the fission yeast *S. pombe* strains, we followed the microdilution method by Clinical Laboratory Standards Institute (CLSI) guidelines, with some modifications as described previously (21, 22). Briefly, strains were inoculated into each well of 96-well microplates with 0.1 mL of YPD medium and varying concentrations of drugs, achieving a cell density of 10^5^ cells/well. The microplates were incubated at 27°C for 48 hours, and then the density of the suspension was determined by measuring optical density (OD) at 530 nm. The MICs of the pathogenic yeasts, including *C. albicans*, *Candida krusei*, *Candida inconspicua*, *Candida tropicalis*, and *Cryptococcus*, were determined according to CLSI protocols M72-A3 guidelines, while the MICs of the filamentous fungi, including *Penicillium* and *Trichoderma,* were determined according to CLSI protocols M38-A2 guidelines with some modifications as described previously (19, 20). In brief, after incubating on PDA plates at 35°C, the pathogenic fungal strains were suspended in the suspension medium and standardized to 2 × 10^3^ CFU/mL by using a hemacytometer. RPMI 1640 medium (10.4 g/L RPMI-1640 with L-glutamine but without bicarbonate, 34.53 g/L MOPS, pH 7.0) was used for suspension cultures of the pathogenic yeasts, while RPMI 1640 medium supplemented with 20 g/L glucose was used for suspension cultures of the filamentous fungi. Then, the fungal suspensions with different concentrations of TBMS1 were filled into each well of 96-well microplates to yield 10^3^ cells/well, and incubated at 35°C for 24–48 hours. The density of the suspension was also determined by measuring OD at 530 nm. For both non-pathogenic yeast *S. pombe* and pathogenic fungi, MIC endpoints were defined as the lowest drug concentrations that inhibited 50% and 80% of the fungal cell growth compared to the growth control. Each MIC was determined in triplicate.

### *S. pombe* cell growth assay

To dynamically assess the inhibitory effect of TBMS1 on *S. pombe*, we conducted a cell growth assay. In short, after pre-culture overnight in YPD at 27°C, the resulting cells were used to inoculate 3 mL YPD cultures with the final cell density of 0.3 at OD 660 nm yielding 10^7^ cells/mL which contained different concentrations of drugs as indicated. The cultures were incubated at 27°C for 7 hours, and the OD 660 was determined every 30 minutes during this period. The experiment was performed in triplicate using independent yeast colonies.

### Transcriptomic analysis

Cells in the early log phase were grown on fresh YPD liquid medium with or without 8 µg/mL TBMS1 for 8 hours and harvested for total RNA extraction. The samples were sent to Sangon Biotech Co., Ltd. (Shanghai, China) for transcriptomic analysis. Raw sequencing data were aligned against the *S. pombe* genome (ASM294v2) with HISAT2 using default settings. DESeq2 R package (Version 1.16.1) was used for analyzing the differentially expressed genes (DEGs) between different groups, providing statistical routines for determining differential expression in digital gene expression data. Genes with an adjusted *q*-value of less than 0.05 and a log2 fold change (log2FC) greater than 2 were considered DEGs. Functional enrichment analysis of DEGs was conducted to identify Gene ontology (GO) categories related to their biological processes, molecular functions, or cellular components. A false discovery rate <0.05 was used as the cut-off value.

### Calcineurin-dependent response element reporter assay

Real-time monitoring of calcineurin-dependent response element (CDRE) reporter activity using the firefly luciferase reporter assay was conducted following the procedure previously described (23). To evaluate the impact of TBMS1 on CDRE activity, the *S. pombe* wild-type cells expressing multicopy reporter plasmid pKB5723 [3 × CDRE::luc(2.2)] were treated with various TBMS1 concentrations. D-luciferin sodium salt monohydrate (50 mM in sterile water as a stock solution; Biosynth Corporation, Switzerland), a substrate for firefly luciferase, was added to the cell suspension at 1:100 dilution. Light emission levels, displayed as relative light units, were determined at 1-minute intervals for 300 minutes using a luminometer (AB-2350; ATTO Co., Tokyo, Japan) at 27°C.

### Mobility shift assay

The expression of GFP-Prz1 in wild-type cells was induced by incubating them in an EMM medium without thiamine at 27°C for 24 hours. Then, cells were treated with TBMS1, CaCl_2_, or FK506 for 30 minutes as indicated in the figure legends. Whole-cell extracts were prepared from these cell cultures as described previously (24). In short, cells were harvested and lysed with 500 µL ice-cold lysis buffer containing 1.85M NaOH and 7.5% β-mercaptoethanol. After processing, 500 µL 50% trichloroacetic acid was added to the suspension and then centrifuged at 4°C, 1 minute, 12,000 rpm. Subsequently, pellets were washed and solubilized in SDS sample buffer (10% glycerol, 5% 2-mercaptoethanol, 2% SDS, 62.5 mM Tris-HCl, and pH 6.8). Then, sodium dodecyl sulfate-polyacrylamide gel electrophoresis (SDS-PAGE) was used to separate the protein extracts (25) with separating acrylamide gels (8%) in a mono/bis ratio of 29.8:0.2. For immunoblotting, purified rabbit polyclonal anti-GFP and mouse anti-human monoclonal antibody β-actin were used as the primary antibodies, and horseradish peroxidase (HRP)‐conjugated goat anti‐rabbit/mouse antibodies were used as the secondary antibodies. β-actin served as a loading control.

### Detection of phosphorylated Pmk1

Cells were grown in YPD medium to mid-log-phase at 27°C and subjected to 1 µg/mL micafungin for 60 minutes treatment or 8 µg/mL TBMS1 for 8 hours treatment. Protein extraction and immunoblot analysis were performed as described previously (26). Phosphorylated Pmk1 was detected by immunoblotting with rabbit monoclonal anti-phospho-p44/42 mitogen-activated protein kinase (MAPK) antibody (Cell Signaling Technology). β-actin served as a loading control. HRP‐conjugated goat anti‐rabbit/mouse antibodies were used as the secondary antibodies.

### Microscopy methods

Various light microscopy techniques, including fluorescence microscopy and differential interference contrast (DIC) microscopy were carried out using a Nikon Eclipse Ni-U microscope equipped with a DS-Qi2 camera (Nikon Instruments Inc., Japan) as described previously (27, 28). Cells in the early log phase were subjected to different conditions for an 8-hour incubation, as indicated in the figure legends. After washing with phosphate-buffered saline (PBS, pH 7.0), cells were stained with Calcofluor White [Sigma-Aldrich (Shanghai) Trading Co. Ltd., China] to visualize the cell wall and septum, and examined microscopically.

For transmission electron microscopy, cells were fixed and embedded essentially as described previously (29). Briefly, early log-phase cells grown in YPD or YPD plus 16 µg/mL TBMS1 at 27°C were collected and fixed with 2% glutaraldehyde EM (Electron Microscopy Science) in 50 mM phosphate buffer, pH 7.2, and 150 mM NaCl (PBS) for 2 hours at 4°C, post-fixed with 1.2% potassium permanganate overnight at 4°C, and embedded in Epon 812 mixture after rinsing and dehydration. Ultrathin sections were stained in 4% uranyl acetate and 0.4% lead citrate. Microscopic examination was performed by using a JEOL JEM-1230 (HITACHI H-7650) transmission electron microscope operating at 80 kV (HITACHI JAPAN), and images were acquired with a Morada CCD Digital Camera and Item software.

### Statistical analyses

Quantitative data were presented as mean ± S.E.M. Statistical analyses were conducted using the SPSS 16.0 software package (SPSS, Inc., Chicago, IL, USA). The one-way analysis of variance (ANOVA) followed by Tukey’s posthoc test was used to assess the statistical significance of results from multiple comparisons. A *P*-value of less than 0.05 was considered statistically significant (*, *P* < 0.05; **, *P* < 0.01; ***, *P* < 0.001).

## RESULTS

### Antifungal activity of TBMS1 against *S. pombe*

**Fig 1:**
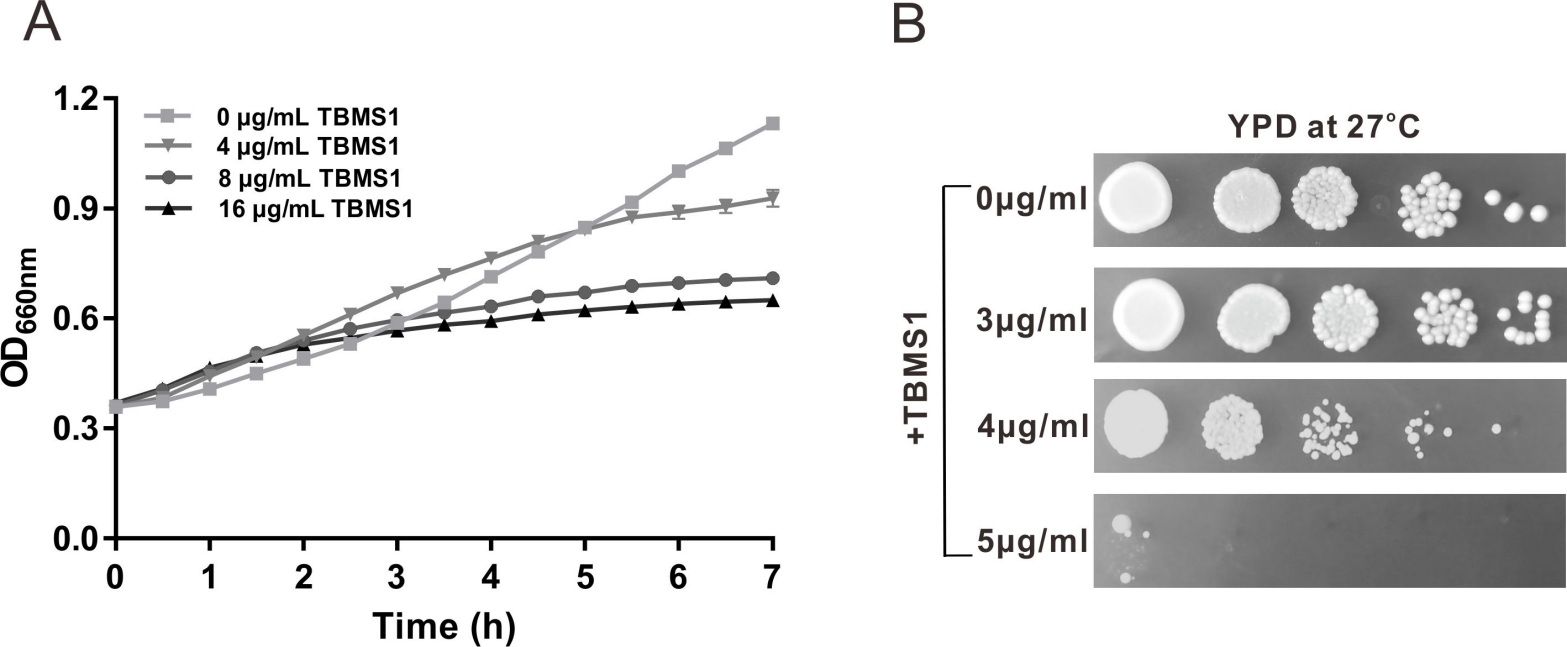
Antifungal activity of TBMS1 against *S. pombe*. (**A**) Wild-type cells were treated with TBMS1 at 0 µg/mL, 4 µg/mL, 8 µg/mL, and 16 µg/mL, respectively. OD660, an index of cell density, was adjusted to 0.3 at the beginning, and then was measured every 30 minutes at 27°C for 7 hours. *n* = 3 for respective time points. (**B**) Fission yeast wild-type cells were spotted on the plates containing different concentrations of TBMS1 as indicated at 27°C for 4 days.

Given that natural products and their extracts have long been important sources of new drugs against infectious diseases (11, 30), we aimed to identify potential antifungal drugs from a collection of natural products using model organism *S. pombe*. Several interesting compounds with strong activities against *S. pombe* were identified using MIC assays, and in this study, we focused on the compound TBMS1, which exhibited the highest antifungal activity against *S. pombe*. As shown in Table 1, TBMS1 displayed good activity against *S. pombe* with a MIC_50_ of 4 µg/mL and a MIC_80_ of 8 µg/mL. To further assess the inhibitory effects of TBMS1 on *S. pombe* dynamically, we monitored its growth over 7 hours while exposing the cells to various TBMS1 concentrations. The results showed that TBMS1 had a significant fungistatic effect in a dose-dependent manner (Fig. 1A). We also tested the effect of TBMS1 on the growth of *S. pombe* wild-type cells on agar plates. As shown in Fig. 1B, TBMS1 at 5 µg/mL significantly suppressed the cell growth, whereas the wild-type cells grew normally at YPD plates (Fig. 1B).

### Transcriptional response of *S. pombe* exposed to TBMS1

**Fig 2:**
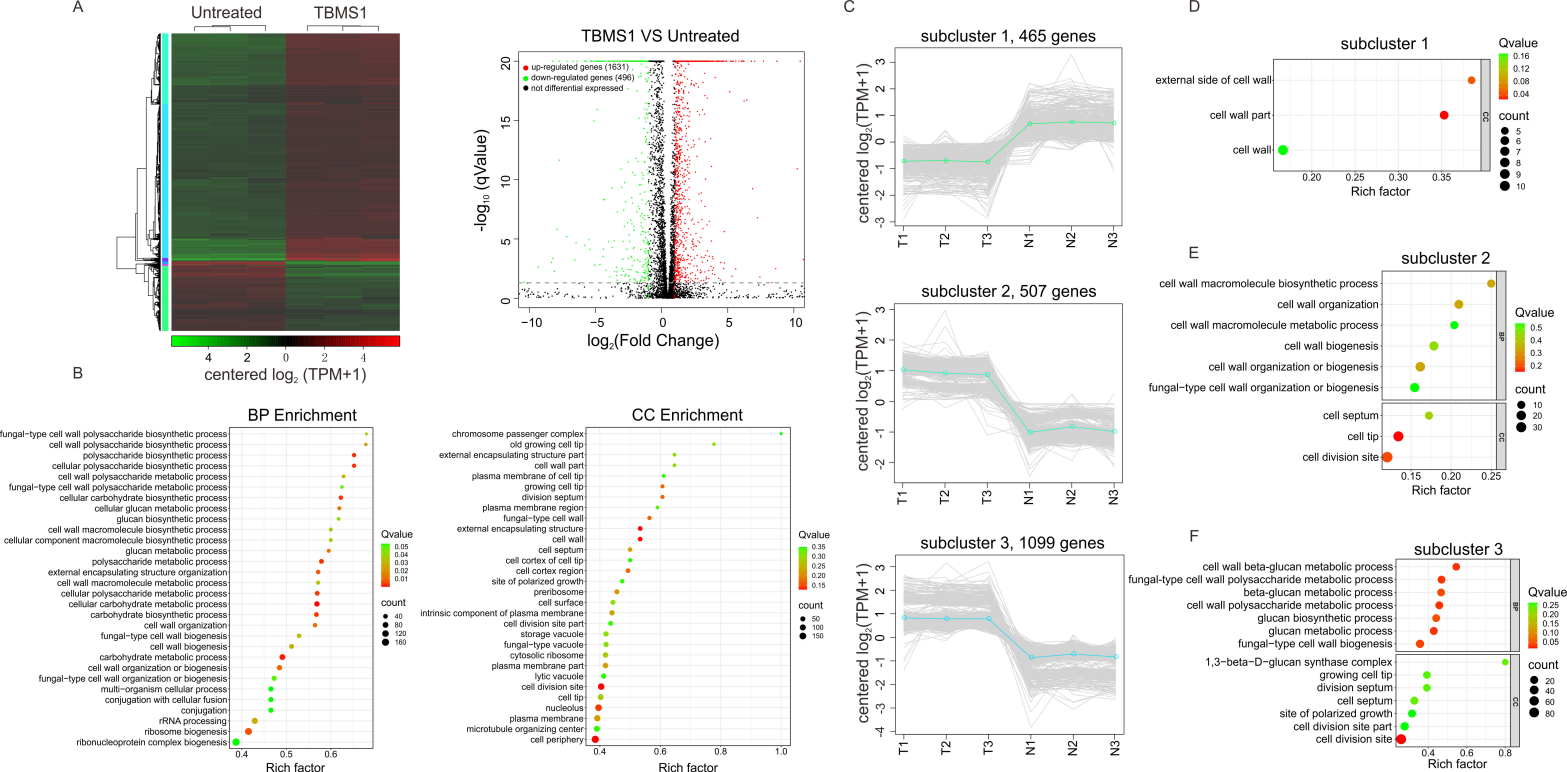
Transcriptomics analysis of *S. pombe* cells treated with TBMS1 relative to untreated cells. (**A**) Heat map and volcano map depicting the clustering of the different samples in terms of DEGs. Green, negatively regulated; red, positively regulated. Color saturation indicates the strength of differential expression. Samples were analyzed in triplicate. (**B**) The top 30 GO terms of DEGs. (**C**) Different subclusters of coregulated genes. (**D–F**) GO analysis of subcluster 1 (**D**), subcluster 2 (**E**), and subcluster 3 (**F**).

To gain insight into the potential molecular basis involved in TBMS1 antifungal activity, we conducted transcriptomics analyses of *S. pombe* cells exposed to a concentration of 8 µg/mL (equivalent to MIC_80_) of TBMS1 relative to untreated cells for 8 hours (see Materials and Methods). TBMS1 treatment had a profound impact on the *S. pombe* transcriptome, resulting in a significant modulation of gene expression. Heat map and Volcano map showed that a total of 2,127 DEGs were identified following exposure to 8 µg/mL TBMS1, including 1,631 up-regulated genes and 496 down-regulated genes (fold change >2 or <−2, *q*-value <0.05, Fig. 2A). We further performed gene ontology (GO) analysis of the DEGs, and the top 30 GO terms in biological process and cellular component categories were displayed in Fig. 2B. Of note, the DEGs were predominantly associated with biological processes related to the function of the fungal cell wall, such as ‘‘cell wall organization or biogenesis,’’ “cell wall macromolecular biogenesis or metabolic process,” “glucan biosynthetic or metabolic process,” and others (Fig. 2B). Consistently, the cellular component categories included “fungal-type cell wall,” “cell septum,” and “cell wall part” (Fig. 2B).

For grouping the DEGs into clusters based on similar expression patterns, we conducted clustering analysis, and generated three different subclusters of coregulated genes (Fig. 2C). Subcluster 1 included a subset of downregulated genes (Fig. 2C, upper panel), whereas both subcluster 2 and subcluster 3 included a subset of upregulated genes (Fig. 2C, lower panel). Importantly, GO analysis showed that several cellular component categories related to cell walls were enriched in subcluster 1. In addition, subcluster 2 genes were majorly involved in the biological processes related to the cell wall, such as ‘‘cell wall organization or biogenesis,’’ and subcluster 3 genes were also mainly associated with the biological processes related to the cell wall, such as “glucan biosynthetic or metabolic process” (Fig. 2D through F). These results suggested that TBMS1 may exert its antifungal activity against *S. pombe* cells by targeting the cell wall.

### TBMS1 stimulates the calcineurin/Prz1 signaling pathway

**Fig 3:**
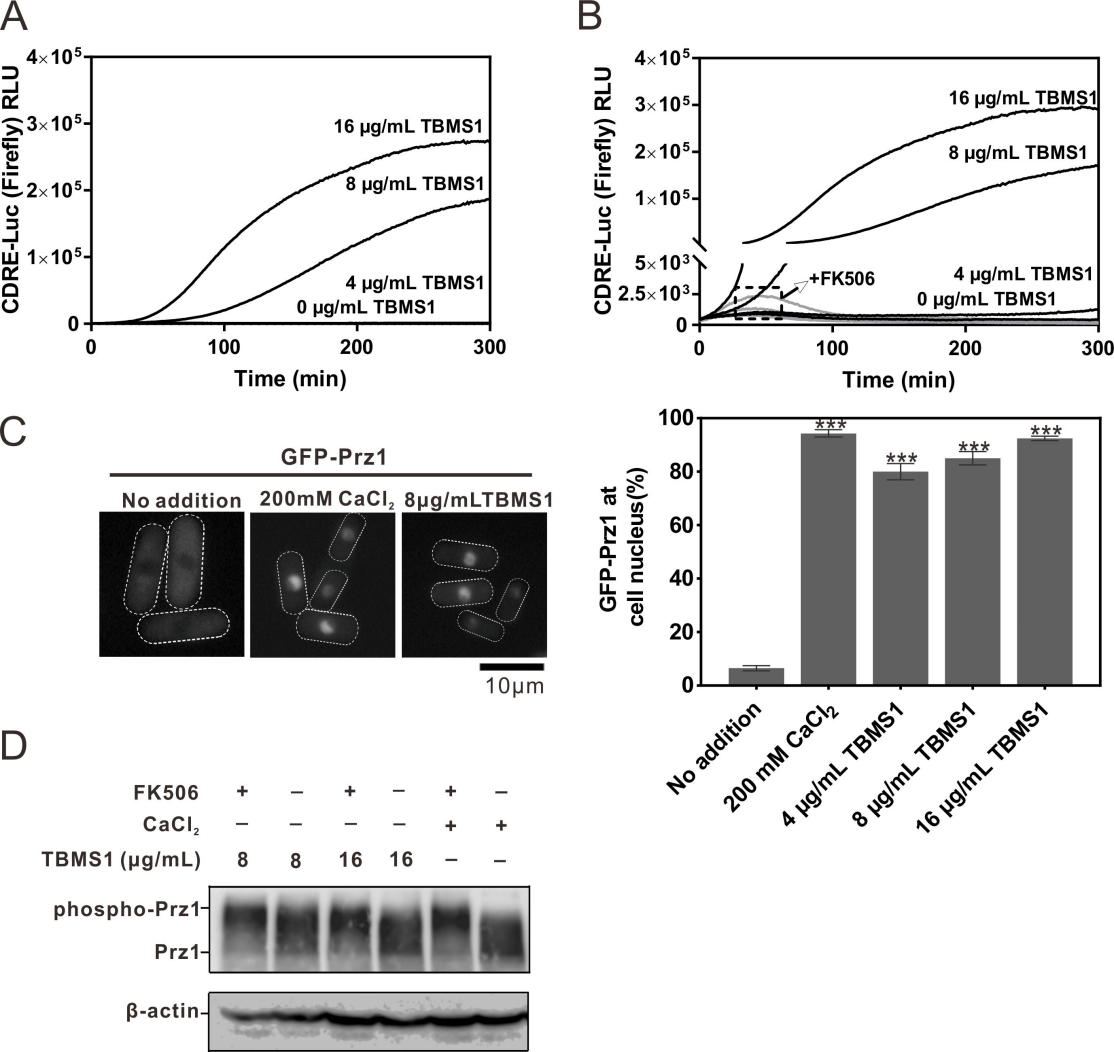
TBMS1 stimulates the calcineurin/Prz1 signaling pathway. (**A**) TBMS1 induced a dose-dependent response of 3 × CDRE::luc(R2.2) reporter. Wild-type cells transformed with the multicopy 3 × CDRE::luc(R2.2) reporter plasmid were incubated with D-luciferin and treated with various concentrations of TBMS1 as indicated and then were analyzed as described in Materials and Methods. Y-axis values are the relative light units of each sample. (**B**) FK506 abolished the increased calcineurin activity caused by TBMS1. Cells were incubated with D-luciferin, treated with FK506 at 0.5 µg/mL, and then treated with TBMS1 as described in A. (**C**) TBMS1 stimulated the translocation of GFP-Prz1 from the cytosol to the nucleus. (Left) Wild-type cells transformed with plasmid GFP-Prz1 were treated with CaCl_2_ or TBMS1. (Right) Quantification of GFP-Prz1 at nucleus in wild-type cells treated as indicated (*n* ≥ 300). ***, *P* < 0.001. (**D**) The mobility shift of GFP-Prz1 protein induced by TBMS1. Wild-type cells expressing GFP-Prz1 were incubated in an EMM medium without thiamine at 27°C with CaCl_2_, TBMS1, or FK506 for 30 minutes. The electrophoretic mobility of the GFP-Prz1 protein was investigated by immunoblotting using a GFP-specific antiserum. β-actin was used as a loading control.

In our previous study, we demonstrated that tamoxifen, a potential antifungal drug targeting the cell wall, induces CDRE transcriptional activity of Prz1 in *S. pombe* cells (9). To investigate whether and how TBMS1 stimulates the calcineurin/Prz1 signaling pathway, we first examined its effects on the CDRE transcriptional activity using the 3 × CDRE::luc (R2.2) reporter system as described previously (23). As shown in Fig. 3A, TBMS1 treatment induced a dose-dependent response of the 3 × CDRE::luc (R2.2) reporter in *S. pombe* cells (Fig. 3A). Furthermore, pretreatment of cells with FK506, a specific inhibitor of calcineurin, abolished the response induced by the addition of various TBMS1 concentrations (Fig. 3B), indicating that the sustained response is specific for calcineurin activity.

Since calcineurin dephosphorylates and activates Prz1, a C_2_H_2_-type zinc finger transcriptional factor, resulting in its translocation from the cytoplasm to the nucleus in *S. pombe* cells (31), we then sought to investigate whether TBMS1 treatment altered the intracellular localization of Prz1. As expected, GFP-Prz1 mainly localized to the cytosol but translocated to the nucleus upon exposure to CaCl_2_ (Fig. 3C). Importantly, the addition of TBMS1 also induced the translocation of GFP-Prz1 to the nucleus (Fig. 3C), further suggesting that TBMS1 treatment enhanced calcineurin activity of *S. pombe* cells.

To further explore the impact of TBMS1 on calcineurin activity, we examined whether TBMS1 influences the mobility shift of Prz1 protein *in vivo* since dephosphorylation of Prz1 by calcineurin led to increased electrophoretic mobility of Prz1 (31). As expected, the mobility shift of GFP-tagged Prz1 was observed when cells were treated with CaCl_2_, whereas the addition of FK506 significantly inhibited the mobility shift (Fig. 3D). Notably, in cells treated with TBMS1, the mobility shift of Prz1 was also clearly observed, and the addition of FK506 to the media significantly inhibited this shift, suggesting that TBMS1 stimulated the dephosphorylation of Prz1 by calcineurin (Fig. 3D).

### TBMS1 caused defects in the cell wall of *S. pombe*

**Fig 4:**
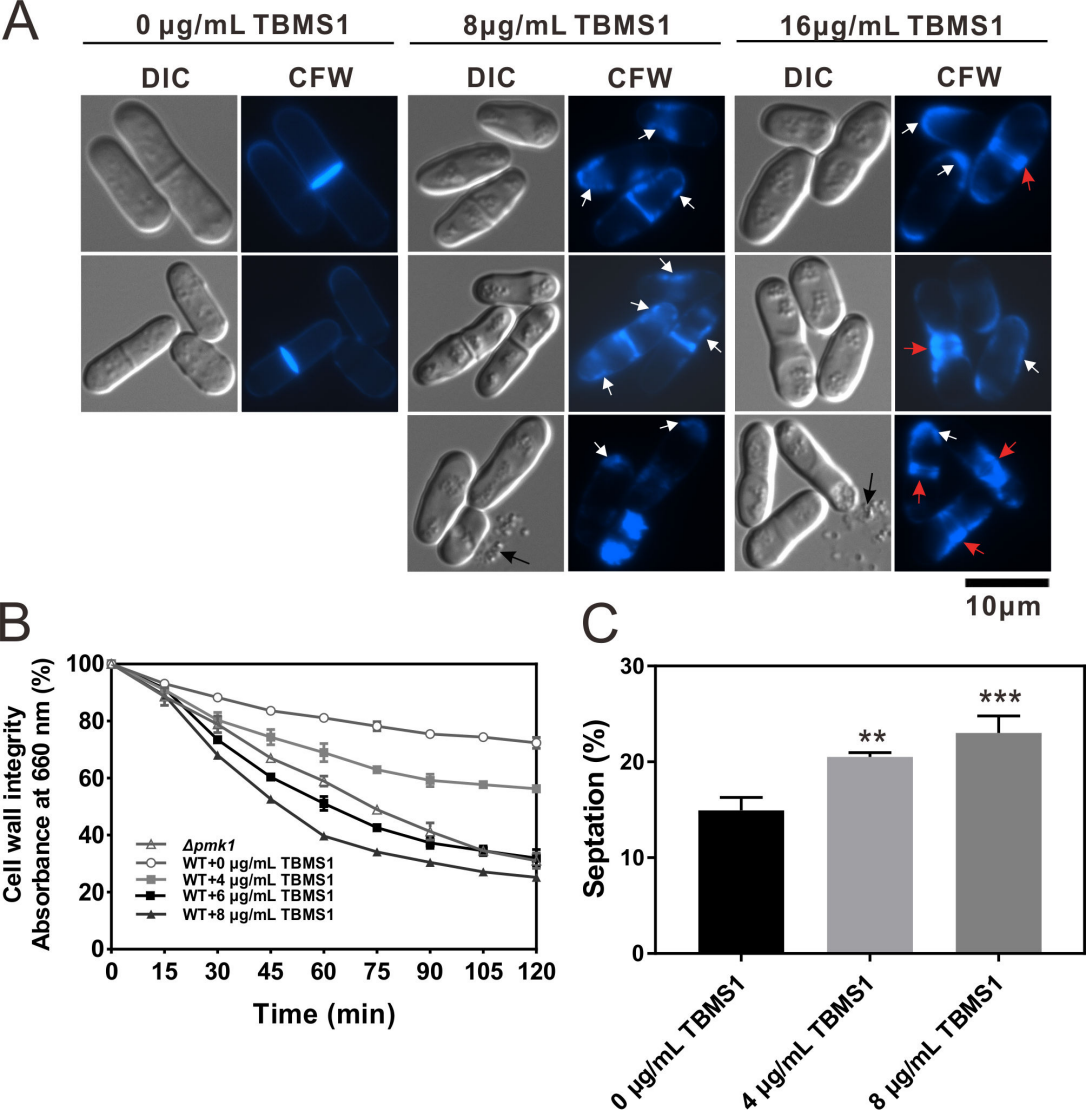
TBMS1 caused defects in the cell wall of *S. pombe*. (**A**) Wild-type cells were grown in a YPD medium containing TBMS1 at 27°C for 8 hours and then stained with calcofluor white and imaged by fluorescence microscopy. Bar, 10 µm. White arrows indicate the abnormal deposits of calcofluor-stained material at cell tips or lateral cell walls, red arrows indicate the thicker septal structure, and black arrows indicate the cytoplasm release. (**B**) TBMS1 markedly enhanced the sensitivity to β-glucanase in a dose-dependent manner. Cells in the log phase were cultured in YPD under different conditions as indicated at 27°C for 8 hours, then washed and incubated with β-glucanase. Cell lysis was monitored by measuring the optical density at 660 nm. (**C**) TBMS1 treatment caused cells an increased septation index in a dose-dependent manner. Septation index (percentage of cells forming a division septum) in wild-type cells with or without the addition of TBMS1 was monitored (*n* ≥ 500). **, *P* < 0.01; ***, *P* < 0.001.

The above findings prompted us to investigate the effects of TBMS1 treatment on the cell wall of *S. pombe*. Then, cells were stained with Calcofluor White to visualize cell wall material. As shown in Fig. 4A, the *S. pombe* cell wall, particularly the septum, is normally the only structure that stains intensely with Calcofluor White. However, TBMS1-treated cells displayed unevenly distributed abnormal deposits of cell wall material at cell tips and along the lateral cell wall (Fig. 4A). In particular, TBMS1 treatment resulted in cells with irregular thickened septa that were brightly stained with Calcofluor White (Fig. 4A). Unlike untreated cells, the TBMS1-treated cells at 16 µg/mL exhibited one prominent thick septal structure in DIC images, whereas Calcofluor White stained the cell middle flanked by two closely spaced structures on each side (Fig. 4A). Notably, cytoplasm release into the medium was occasionally observed in TBMS1-treated cells in DIC images (Fig. 4A), possibly due to the weakened cell wall caused by TBMS1.

We also evaluated the impact of TBMS1 treatment on the resistance of wild-type cells to cell wall damage using β-glucanase sensitivity assay as described previously (9). The Pmk1 deletion was used as a positive control since the *pmk1*^+^ gene encodes a MAPK controlling the cell wall integrity of fission yeast *S. pombe* (17). As shown in Fig. 4B, TBMS1 treatment significantly increased the sensitivity of wild-type cells to β-glucanase dose-dependently (Fig. 4B). Since a high septation index (the percentage of cells in a population containing a septum) can reflect an aberrant cell wall (32), we investigated the effects of TBMS1 treatment on the septation index. As shown in Fig. 4C, the TBMS1-treated cells exhibited an increased septation index in a dose-dependent manner (Fig. 4C). These results suggested that TBMS1 treatment resulted in cell wall defects.

**Fig 5:**
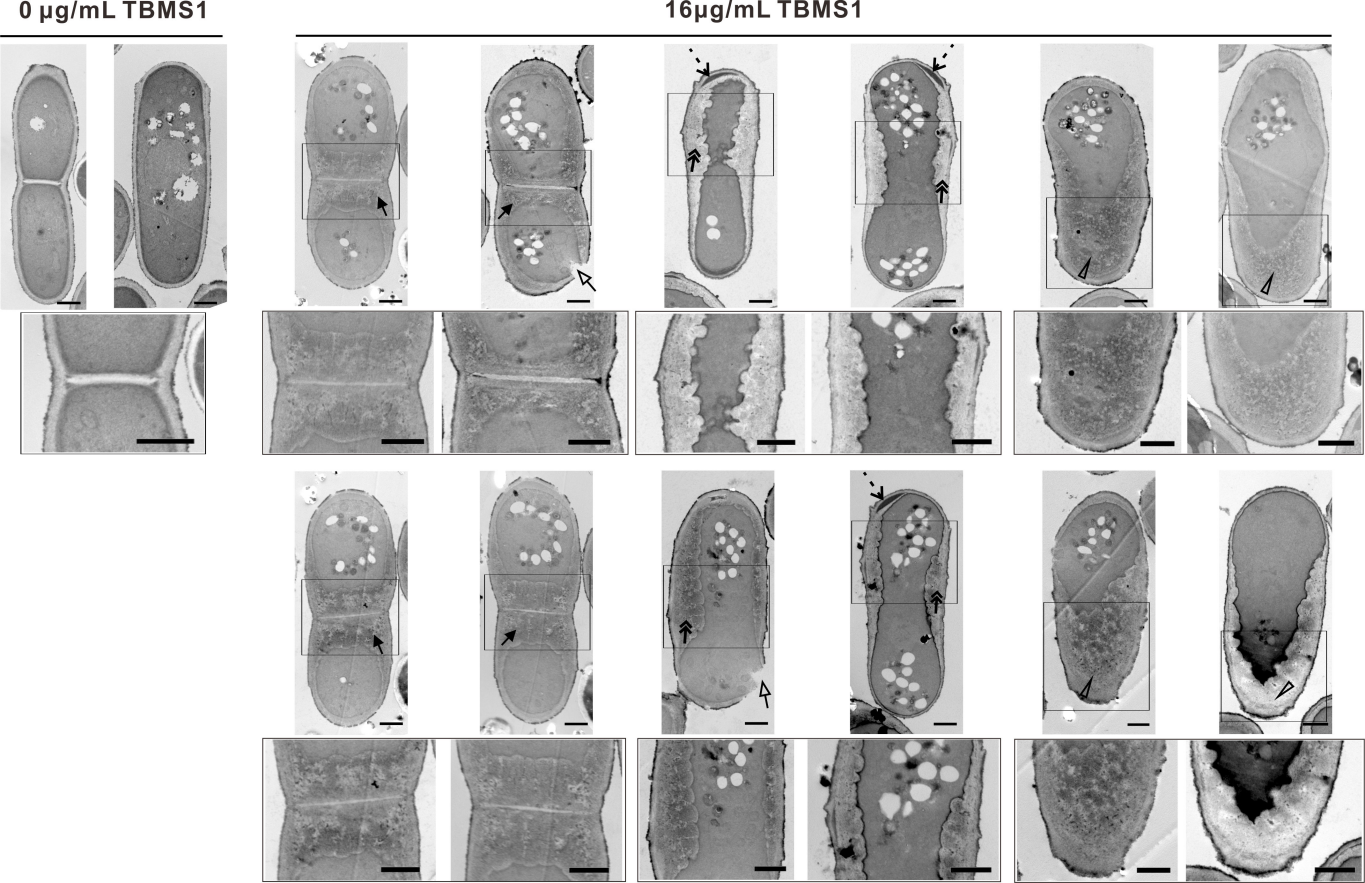
Transmission electron microscopy images of *S. pombe* treated with or without TBMS1. The up panel shows the TEM images of the whole cell. Bar, 1 µm. The boxed areas in the images of the up panel enlarged to the bottom of the original images. The bottom panel displays the detail of the disorganized, thicker cell wall, and division septum. Black arrows indicate the thicker septum, double arrows indicate the thicker lateral cell wall, triangles indicate the thicker cell tip, dotted arrows indicate the lacunae appearing in the remedial layer, and open arrows indicate the cell wall breakage. Bar, 1 µm.

To further confirm this conclusion, we sought to examine the ultrastructure of the cell wall upon exposure to TBMS1 using transmission electron microscopy (TEM). We analyzed cells with or without a division septum that were longitudinally cut. As shown in Fig. 5, the control cells (0 µg/mL TBMS1) exhibited a uniform cell wall structure and a typical three-layered structure in complete septa that consisted of an electron-transparent primary septum in the middle flanked by electron-dense secondary septum on each side (Fig. 5), as previously described (29, 33). Strikingly, the TBMS1-treated cells exhibited extremely aberrant cell wall architecture. In the TBMS1-treated cells, the cell wall and the septum were tremendously thicker and looser than that of the untreated cells (Fig. 5). The aberrant cell walls could even be multilayered, and disorganized accumulation of wall materials could be observed. The TBMS1-treated cells displayed a remedial internal cell wall layer and a remedial secondary septum layer, which extended along the cell wall and the septum, and lacunae were frequently formed in the remedial layer (Fig. 5). These changes may respond to the Calcofluor stained materials shown in Fig. 4A. In the TBMS1-treated cells, cell wall breakage was often observed (Fig. 5), which may lead to cytoplasm release. Thus, these results further reinforced the conclusion that TBMS1 caused structural defects in the cell wall of *S. pombe*.

### TBMS1 activated Pmk1-mediated cell wall integrity pathway

**Fig 6:**
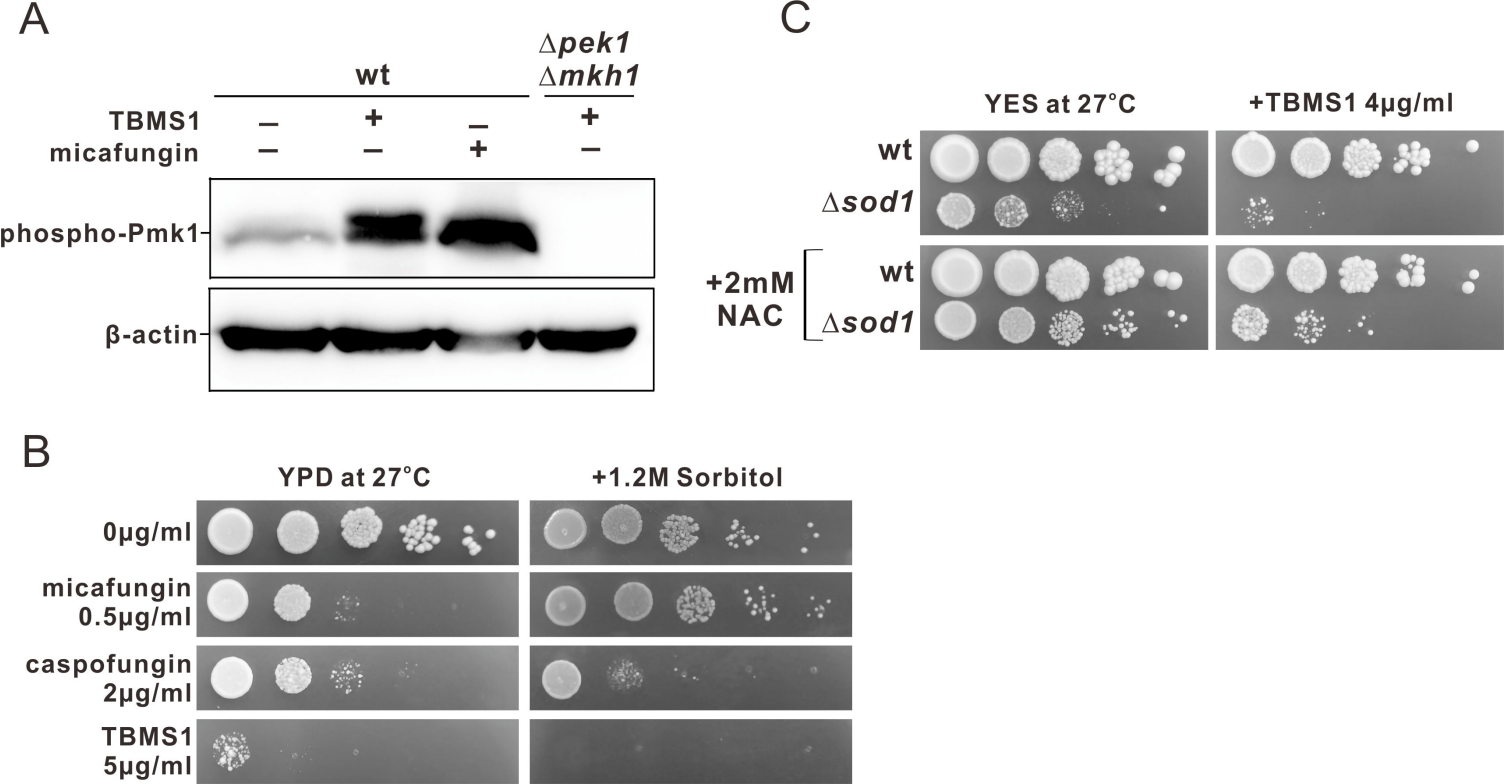
TBMS1 activated Pmk1-mediated cell wall integrity pathway and oxidative stress might be involved in the antifungal action of TBMS1. (**A**) TBMS1 treatment significantly increased Pmk1 phosphorylation. Wild-type cells and ∆*pek1*∆*mkh1* cells were grown in YPD to mid-log-phase at 27°C and subjected to micafungin or TBMS1 treatments as indicated. Phosphorylated Pmk1 was detected by immunoblotting with an anti-phospho-p44/42 MAPK antibody. (**B**) Sorbitol failed to suppress the TBMS1 sensitivity of the *S. pombe* cells. Wild-type cells were spotted onto the plates as indicated and then incubated for 4 days at 27°C. (**C**) Sod1 deletion cells exhibited sensitivity to TBMS1, and this sensitivity was significantly suppressed in the presence of N-acetylcysteine (NAC). Wild-type (*h^−^ leu1-32 ade6-M216*) or Sod1 deletion (*h^−^ leu1-32 ura4-D18 ade6-M216 sod1::ura4^+^*) cells were spotted onto the plates as indicated and then incubated for 4–5 days at 27°C.

Since activation of MAPKs by TBMS1 has been described in mammalian cells (34), and the fungal cell wall integrity is controlled by a MAPK pathway (35), we then determined whether TBMS1 treatment could stimulate the phosphorylation of Pmk1, the core member of the cell wall integrity pathway in *S. pombe*. The ∆*pek1*∆*mkh1* cells were used as a control since Pek1 and Mkh1 activate and act upstream of Pmk1 in the MAPK signaling pathway (36, 37). As expected, cell wall stress with 1 µg/mL micafungin for 60 minutes increased the level of Pmk1 phosphorylation (Fig. 6A), as previously reported (38). Importantly, TBMS1 treatment for 8 hours significantly increased Pmk1 phosphorylation in wild-type strains, but not in ∆*pek1*∆*mkh1* strains (Fig. 6A), indicating that the Pmk1-mediated cell wall integrity pathway was activated in response to TBMS1.

### Oxidative stress might be involved in the antifungal action of TBMS1

Since defects in the cell wall can sometimes be compensated for by osmotic stabilization of the medium with sorbitol, an osmotic protector (39), we then examined whether the inhibitory effect of TBMS1 on the growth of *S. pombe* cells is reduced by the addition of sorbitol to the medium. Caspofungin and micafungin, inhibitors of 1,3-β-D-glucan synthase, were used as a control. Results showed that sorbitol suppressed the micafungin sensitivity of the cells, but failed to suppress the caspofungin sensitivity and TBMS1 sensitivity of the cells (Fig. 6B). Probably caspofungin sensitivity and TBMS1 sensitivity were not caused only by the alterations of cell wall. Since some antifungals, such as clotrimazole, have been shown to promote strong oxidative stress on yeast cells (40), we also investigated whether oxidative stress was associated with the antifungal action of TBMS1. We found that cells deficient in Sod1, a superoxide dismutase that functions in antioxidant systems, exhibited sensitivity to TBMS1, and this sensitivity was significantly suppressed in the presence of the antioxidant N-acetylcysteine (NAC) (Fig. 6C). This result suggested that oxidative stress might also be involved in the antifungal action of TBMS1.

### The antifungal activity of TBMS1 against diverse pathogenic fungi

To further assess the antifungal activity of TBMS1, we performed the MIC assays to determine the *in vitro* activity of TBMS1 toward a variety of clinical isolates of pathogenic fungi (Table 1). Fluconazole was used as a control since the azole class of antifungals serves as a frontline of defense against many different types of invasive fungal infections. As shown in Table 1, TBMS1 exhibited good *in vitro* activity against various *Candida species*, including *C. krusei* (MIC_80_, 32 µg/mL), *C. inconspicua* (MIC_80_, 32 µg/mL), *C. albicans* (MIC_80_ range of 32 to 64 µg/mL), *C. tropicalis* (MIC_80_, 64 µg/mL). Other fungi against which TBMS1 was highly active included *Cryptococcus*(MIC_80_, 64 µg/mL), *Penicillium* (MIC_80_ range of 32 to 64 µg/mL), and *Trichoderma* (MIC_80_, 32 µg/mL) (Table 1).

**Fig 7:**
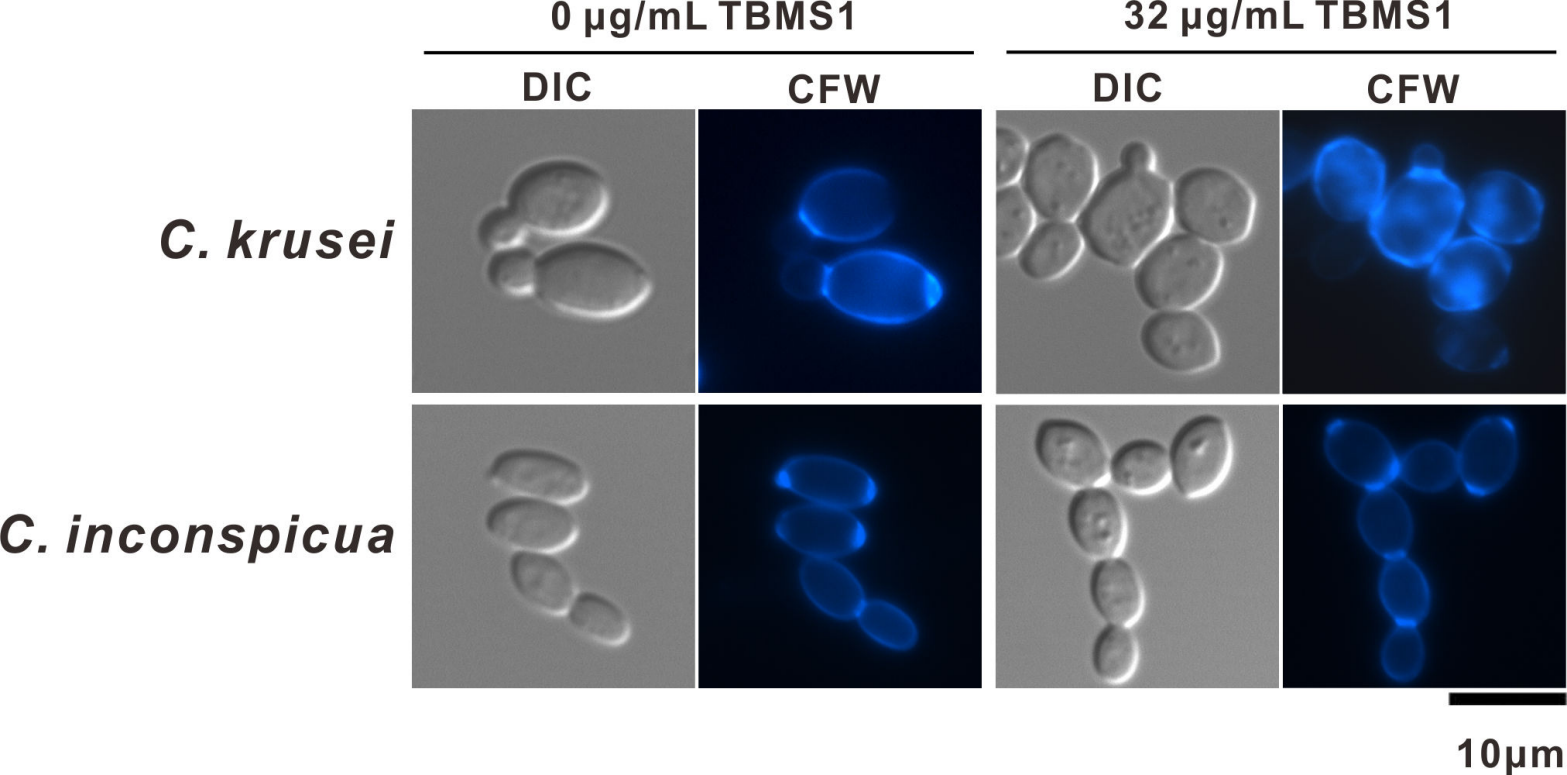
Effects of TBMS1 on the cell wall of pathogenic fungi. Clinical isolates of pathogenic fungi, *C. krusei* and *C. inconspicua*, were grown in YPD culture with or without the addition of TBMS1 at 27°C for 20 hours, respectively, and then stained with calcofluor white and imaged by fluorescence microscopy. Bar, 10 µm.

Since our data described above indicated that TBMS1 disrupted the cell wall in *S. pombe*, we hypothesized that it might interfere with the cell wall in pathogenic fungi as well. To test this hypothesis, we selected two pathogenic fungi with good TBMS1 activities, *C. krusei* and *C. inconspicua*, and examined the effects of TBMS1 treatment on their cell walls and septa using calcofluor white staining. As shown in Fig. 7, for both *C. krusei* and *C. inconspicua*, the fluorescent dye stained strongly the bud necks and scars, and less strongly but uniformly the lateral cell wall (Fig. 7). However, in the TBMS1-treated *C. krusei*, abnormal intensive fluorescence was frequently observed in the lateral cell wall as well as cytoplasm (Fig. 7), similar to that of *S. pombe* shown in Fig. 4A. In addition, TBMS1 treatment led to *C. inconspicua* forming large aggregates that did not separate properly, and additionally fluorescent connections were evident between the aggregate cells (Fig. 7), a phenomenon not observed in the control cells.

## DISCUSSION

Nowadays, fungal infections are a global concern affecting millions of people. The development of novel antifungals to combat life-threatening fungal diseases is urgent, and it is currently one of the most significant challenges in scientific research. The essentiality of the cell wall for fungal survival together with the fact that their components are absent in mammals make this structure an appealing target for therapeutic interventions against fungal infections. In this work, we sought to identify novel antifungal candidates for combating the fungal disease and found that compound TBMS1 inhibited the fungal growth and caused damage to the cell wall, adding a new mode of antifungal action. To our knowledge, this is the first report of the antifungal action of TBMS1 and the underlying mechanism.

Recent progress in chemical genomics research has significantly contributed to our understanding of drug mechanisms and the gene networks associated with the side effects and/or efficacy of various drugs (5). Particularly, the fission yeast *S. pombe* has promoted a thorough understanding of many biological processes involved in drug actions and has been serving as an excellent model system for elucidating the mechanisms of drug actions (21, 22, 41, 42). Despite some variations in cell wall composition among fungal species, the fundamental structure of the fungal cell wall is conserved. Like pathogenic fungi, *S. pombe* is protected by a multilayer cell wall composed of alpha and beta glucans, along with various glycoproteins (43, 44). This flexible and dynamic structure responds to and protects the cell from environmental stresses while accommodating morphogenesis. Consequently, there are exciting opportunities to develop antifungal drugs targeting cell wall components using *S. pombe*. In this study, we found that TBMS1 strongly inhibited the growth of yeast *S. pombe* by disrupting the cell wall. Moreover, TBMS1 exhibited various levels of inhibitory activities against several pathogenic fungi isolated from clinical fungal infections. Most importantly, TBMS1 affected both non-pathogenic yeast *S. pombe* and pathogenic fungi in a similar manner, emphasizing the significance of using *S. pombe* to explore the detailed mechanisms underlying the antifungal action of TBMS1.

Our present study provides several lines of evidence to support the notion that compound TBMS1 exerts its antifungal activity by targeting cell walls. First, transcriptomic data revealed that TBMS1 affected the processes of cell wall organization or biogenesis, since TBMS1 treatment induced significant differential expression of many genes associated with those functions. Consistently, it has been reported that treatment with some antifungal drugs can trigger changes in the expression of cell wall biosynthetic enzymes, structural proteins, and remodeling enzymes (45, 46). Second, TBMS1 treatment significantly increased the sensitivity of *S. pombe* cells to β-glucanase, a key enzyme in cell wall synthesis and remodeling. Third, our data that TBMS1 induced a CDRE response, nuclear accumulation of GFP-Prz1, as well as dephosphorylation of Prz1 reinforces the idea that TBMS1 treatment caused defects in cell wall integrity since calcineurin is thought to regulate the synthesis of 1,3-D-glucan and is involved in cell wall integrity (47). It has been reported that the cell wall integrity pathway is a critical signaling cascade responsible for regulating cell wall growth and homeostasis, which is necessary for adapting to cell wall perturbing conditions (48, 49). It is possible that TBMS1 stimulated the calcineurin pathway, which functionally compensated for the defective cell wall integrity caused by TBMS1 exposure. Fourth, Calcofluor White staining and TEM analysis demonstrated that TBMS1 caused striking abnormalities in the cell walls and septum structures, which led to the rupture of the cell wall and the release of cytoplasm, ultimately causing cell death. Consistently, in comparison to the control cells, the TBMS1-treated cells showed a significantly higher septation index, potentially due to difficulties in splitting the abnormal septal structures, which hinders proper cell separation. It is essential to understand the mechanisms underlying the action of compounds since it will help to develop improved second-generation compounds with better pharmacological properties. Our present data that TBMS1 not only caused cell wall structural defects but also impacted the cell wall integrity signaling, may provide important guidance in designing and developing new cell wall-targeting agents.

In general, an accurate balance between cell wall biosynthesis and degradation has to be maintained to permit fungal cell growth and separation, while aberration in cell wall homeostasis has a large impact on fungal cell survival, leading to cell wall disruption and cell death (50). Whether TBMS1 directly affects cell wall biosynthesis or degradation for its antifungal action remains unknown. Additionally, we cannot rule out the possibility that TBMS1 interferes with the vesicle transport, which could impair cell wall synthesis and remodeling. The exact molecular target of TBMS1 for its antifungal action by disrupting the cell wall warrants further investigation.

In summary, we have identified the compound TBMS1 that exhibits potent antifungal activity by targeting cell walls. Our data presented here not only provide evidence for TBMS1 serving as an antifungal candidate but also provide a mechanistic basis for future studies designed to systematically optimize the antifungal activities of this candidate scaffold. Our results indicate that TBMS1 represents a potentially important scaffold for the development of new therapies to combat life-threatening fungal diseases.

## Data Availability

The transcriptome data reported in this paper have been deposited in the NCBI Sequence Read Archive database (BioProject accession no. PRJNA1089978). All data necessary for confirming the conclusions of the article are presented within the article, figures, and tables.
